# Our Experience With Extracorporeal Irradiation and Reimplantation of the Irradiated Bone for the Reconstruction of Bone Defects Following Tumor Resection

**DOI:** 10.7759/cureus.52853

**Published:** 2024-01-24

**Authors:** Nageswara Rao Kancherla, Sreenija Paruchuri, Bodla Arvind, Shravan Peddamadyam, Srikanth Eppakayala, Nagesh Cherukuri

**Affiliations:** 1 Department of Orthopedics, Nizam's Institute of Medical Sciences, Hyderabad, IND; 2 Department of Orthopedics, Kamineni Institute of Medical Sciences, Narketpally, IND; 3 Department of Orthopedics and Trauma, All India Institute of Medical Sciences, Jodhpur, Hyderabad, IND

**Keywords:** bone defect, tumor resection, reconstruction after tumor resection, extracorporeal irradiation, bone tumors

## Abstract

Background: With tremendous improvement in the survival of patients with malignant bone tumors, there is a greater emphasis on the functional outcome and durability of reconstruction following tumor resection. Tumor resection and extracorporeal irradiation (ECI) followed by reimplantation of the irradiated bone in malignant bone tumors is drawing popularity across various centers. In this study, we aim to put forward our experience with ECI, the outcomes achieved, and the complications faced by us.

Methods: This is a prospective study conducted in patients with malignant and locally aggressive bone tumors who underwent ECI at our center. A total number of 20 patients were selected for the study and followed up for a mean duration of 32.5 months (maximum duration: 58 months, minimum duration: eight months). Orthopedic outcome was measured using the Enneking score. We assessed for local recurrence, functional outcome, union, and complications during the follow-up.

Results: During the course of follow up, local recurrence was seen in two patients. The mean MSTS score of the remaining patients was found to be 23.6. Complications seen included limb length discrepancy, surgical site infection, and graft lysis.

Conclusion: Tumor resection and ECI is an effective procedure for biological reconstruction which gives satisfactory functional outcomes. In spite of certain complications, patients expressed satisfaction with the overall outcome of the procedure.

## Introduction

Wide en bloc resection of malignant bone tumors results in a sizable bone defect that needs to be reconstructed to achieve a satisfactory functional outcome. Various mechanical and biological methods of reconstruction are available to fill the gap after wide tumor resection. Biological methods of reconstruction provide a standard option for malignant bone tumor surgery as they provide permanent structural repair and seldom require further revision procedures [1]. Various biological reconstruction options are available including vascularized or non-vascularized fibular autografts, allograft, autograft-allograft composites, and distraction osteogenesis [2]. Fibular grafting is associated with complications including fractures, nonunion, donor site morbidity involving the remaining “good limb,” and failure of vascular anastomosis [3]. The use of bone allografts may be limited by their availability in developing countries, risk of infection transmission, and nonunion [4]. Though distraction osteogenesis provides a bone that is biomechanically strong and durable, the procedure is relatively time-consuming, demanding, and not patient-friendly [3]. A further promising autologous treatment option, therefore, is the reimplantation and fixation of the tumor-bearing bone segment after destroying the tumor cells. This can be achieved by treatment with liquid nitrogen, pasteurization, microwave radiation, autoclaving, or extracorporeal irradiation (ECI) [5,6]. ECI is an innovative technique where the resected bone segment itself, after irradiation, acts as an anatomically size-matched bone graft to fill the resultant bone defect. It consists of wide en bloc resection of the tumor-bearing bone segment, removal of the tumor and soft tissues from the bone, irradiation, and reimplantation back in the body with a fixation device [7]. The dose of radiation should be enough to kill all the tumor cells, thus, leaving no risk of local recurrence or radiotherapy-induced malignancy [8]. In addition to providing a three-dimensionally matched graft, this technique also removes the need for bone banks, risks of infection transmission, and immunological problems [9]. Lately, it has become a mainstream technique in many cancer centers across the world. Though it consumes more operating time for the process of irradiation, it puts less financial burden on the patient [10]. Since it was first reported by Spira and Lubin in Australia (1968) [11,12], it has widely gained popularity and has been tested at multiple centers with respect to the oncological outcomes, functional outcome, radiation dose, complications, site of resection, etc. As the majority of patients with malignant bone tumors are children and adolescents with high functional demands and growth potential, this biological method of reconstruction has proven to be a useful technique for limb salvage, especially in low socioeconomic conditions. In this study, we would like to report our experience of the aforementioned procedure with emphasis on the complications that we faced.

## Materials and methods

This is a prospective study conducted in the Department of Orthopedics, Nizam’s Institute of Medical Sciences, Hyderabad, after obtaining clearance from the institutional ethics committee (1108/2021). From 2019 to 2023, 20 cases were selected for tumor resection and ECI followed by reimplantation of the irradiated bone. We selected cases where the metallic prosthesis was not feasible, growing children in whom biological reconstruction is perceived better, and where the articular surface was not involved by the tumor. After thorough clinical examination and appropriate investigations, under the informed and written consent of the patient/guardian, the procedure was performed, and the patient was kept under regular follow-up. One patient who was lost to follow-up after the surgery was excluded. Hence 19 patients included in the study were followed up and observed regularly(n=19). Data analysis was done in November 2023.

Inclusion criteria

Patients who underwent tumor resection and ECI for biopsy-proven malignant and locally aggressive musculoskeletal tumors were included in the study.

Exclusion criteria

Patients who were lost to follow-up are excluded from the study.

Preoperative assessment

After the establishment of a histopathological diagnosis of bone tumors, patients with malignant and locally aggressive bone tumors were selected. Patients with malignant bone tumors requiring chemotherapy received neoadjuvant chemotherapy before surgery as per the protocols of the medical oncologist. A post-chemo MRI was obtained and compared to the initial MRI to evaluate the chemotherapy response and to plan the surgical resection. Relevant blood investigations were performed. A CT angiography of the involved extremity was obtained to look for major feeder vessels and to get an idea of the overall vascularity of the tumor. Preoperative embolization of the tumor feeder vessel was performed wherever deemed necessary.

Surgical procedure

**Figure 1:**
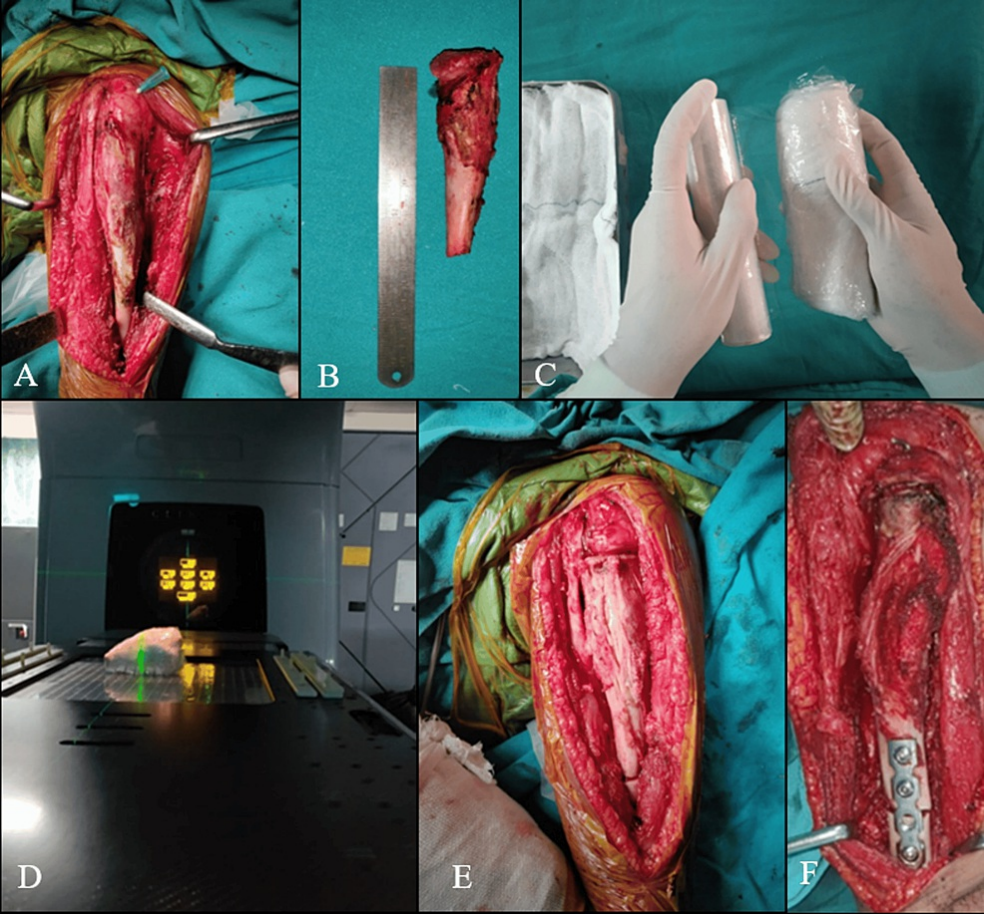
Series of images depicting the surgical procedure in a case of Ewings sarcoma of humerus in a five-year-old girl (A) Surgical exposure. (B) Resected tumor bearing bone segment. (C) Specimen packed to prevent airpockets. (D) Irradiation of the specimen. (E) Specimen placed back into the defect. (F) Internal fixation.

General or combined spinal with epidural anesthesia is given based on the tumor location. After positioning and draping, the main artery supplying the involved limb is exposed and clamped for vascular control. With the help of preoperative MRI, adequate margins were taken and en bloc resection of the tumor was performed, supplemented by an intraoperative frozen section to confirm tumor-free margins. The resected bone was sterile packed in antibiotic-soaked mops and wrapped in a cling drape. The final dimensions of the packed specimen were communicated with the radiation oncologist. The specimen is subjected to a 50 Gy dose of radiation (photon x-rays) as a single fraction from a linear accelerator. The process of irradiation, including transport to and from the linear accelerator consumed around 40 minutes. After irradiation, the specimen was transported back to the operation theatre where, if required, the exuberant growth from the tumor was trimmed. It is then fixed into the bone defect using appropriate internal fixation devices. Supplementation using a vascular or avascular fibular graft was performed in most cases (Figures 1A-1F).

Follow up

After complete wound healing and suture removal, the 19 patients were followed up once a month for the first year and then once in three months for the subsequent years. Radiographs of the chest and operated limb were obtained at every follow-up. The mean duration of follow-up in our study was 32.5 months, with a minimum follow-up of eight months and a maximum follow-up of 58 months. During follow-up, we looked for local recurrence, distant metastasis, complications, and functional outcome. Local recurrence was ruled out by performing clinical examination and plain radiographs. A chest radiograph was used to evaluate lung metastasis. The Enneking (MSTS) score was used to evaluate the overall functional outcome of the procedure [13].

## Results

Twenty patients underwent ECI at our center in the years 2019 to 2023 (Table 1).

**Table 1:** Summary of 20 cases who underwent extracorporeal irradiation at our center

S.NO	AGE	SEX	HISTOPATHOLOGY	ANATOMICAL LOCATION	FOLLOW UP	COMPLICATIONS	TIME TO UNION	MSTS SCORE
1	11	M	Ewings sarcoma	Tibial diaphysis	6 months	Local recurrence leading to amputation	4 months	-
2	9	F	Ewings sarcoma	Proximal humerus metadiaphysis	58 months	Limb length discrepency	4 months	24
3	43	M	Adamantinoma	Distal tibia metadiaphysis	50 months	nil	7 months	28
4	12	M	Osteosarcoma	Distal tibia metaphysis	52 months	Dermatitis	4 months	26
5	10	M	Telengiectatic osteosarcoma	Distal femur metaphysis	49 months	Nil	3 months	26
6	7	M	Ewings sarcoma	Proximal humerus metadiaphysis	47 months	Graft lysis	3 months	21
7	16	F	Ewings sarcoma	Proximal radius metadiaphysis	3 months	local recurrence leading to amputation	-	-
8	18	M	Ewings sarcoma	Femur diaphysis	46 months	surgical site infection	7 months	26
9	5	F	Ewimgs sarcoma	Humerus diaphysis	36 months	Limb length discrepency	4 months	27
10	9	M	Ewings sarcoma	Femur diaphysis	38 months	Nil	5 months	28
11	25	M	Osteosarcoma	Proximal Tibial Metadiaphysis	20 months	Surgical site infection leadng to amputation	-	-
12	35	M	Secondary osteosarcoma	Proximal humerus metaphysis	28 months	Surgical site infection	6 months	18
13	20	M	Giant cell tumor	Distal tibia epimetaphysis	-	lost to follow up	-	-
14	45	F	Osteoblastoma	Femur diaphysis	18 months	nil	6 months	30
15	17	F	Giant cell tumor	Distal tibia epimetaphysis	16 months	Surgical site infection	Non union	19
16	45	M	Fibrocartilagenous mesenchymoma	Pelvis	24 months	nil	4 months	20
17	20	F	Giant cell tumor	Proximal humerus epimetaphysis	8 months	Nil	5 months	22
18	40	M	Chondrosarcoma	Distal humerus metadiaphysis	9 months	nil	4 months	29
19	50	M	Phosphaturic mesenchymal Tumor	Proximal Tibia Epimetaphysis	20 months	nil	5 months	28
20	31	M	Giant cell tumor	Proximal Tibia Epimetaphysis	21 months	nil	5 months	26

**Figure 2:**
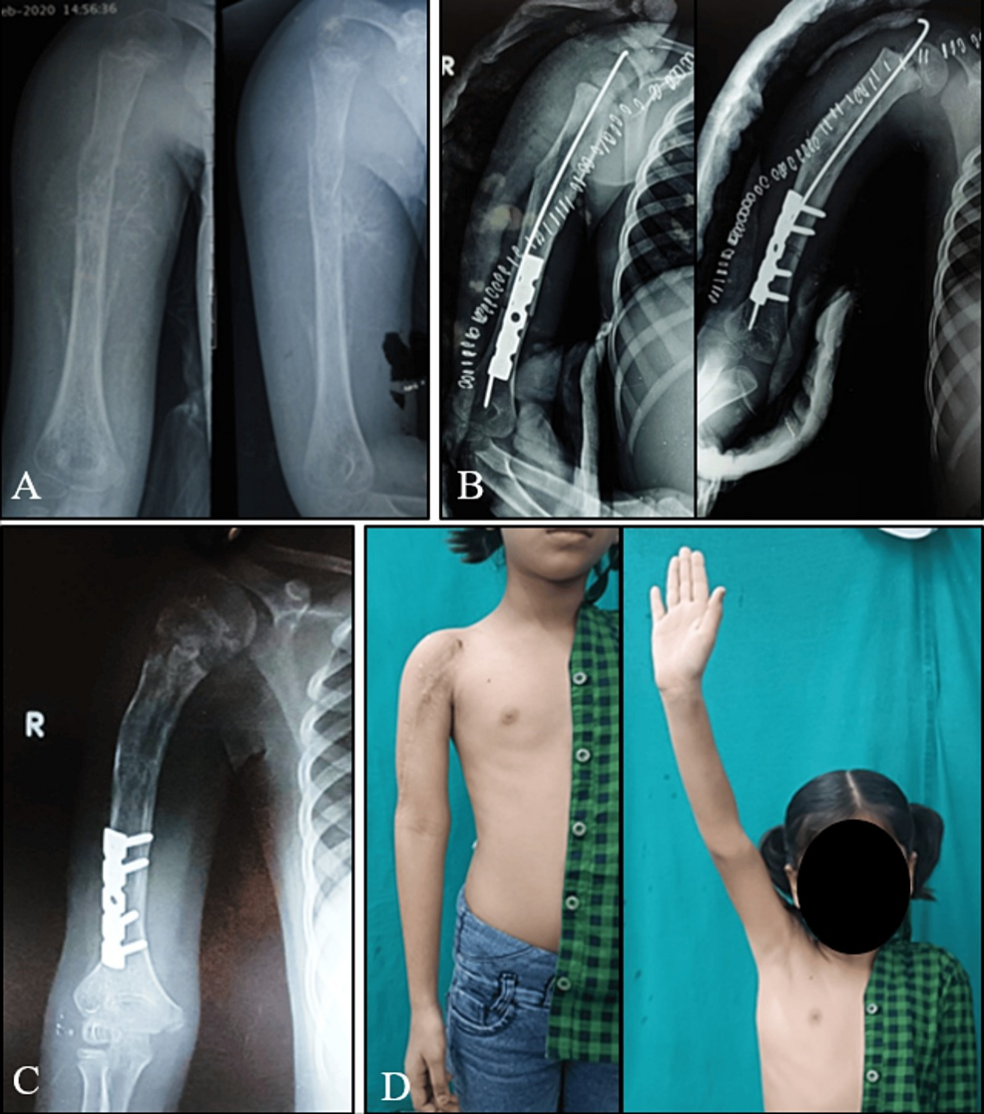
Follow-up images of a five-year-old girl with Ewings sarcoma of humerus (A) Preoperative radiograph, (B) immediate post operative radiograph, (C) follow-up radiograph at 34 months, (D) shoulder function at 34 months.

One patient (case 13) lost to follow-up immediately after the surgery and was therefore excluded from our study. The remaining 19 patients were followed up regularly (n=19). Three patients underwent amputation during the course of the study period. These three cases were excluded while calculating mean follow-up and MSTS score. The minimum duration of follow-up was eight months and the maximum duration was 58 months with a mean follow-up of 32.5 months. Out of the 19 cases, 12 patients had a histological diagnosis of malignant bone tumors (Seven - Ewings sarcoma, Four - Osteosarcoma, One - Chondrosarcoma) while seven patients had locally aggressive neoplasms (Three - Giant cell Tumor, One - Adamantinoma, One - Fibrocartilagenous mesenchymoma, One - phosphaturic mesenchymal tumor, One -Osteoblastoma). The tumor location was in the upper limb in seven patients, the lower limb in 12 patients, and pelvis in one patient. In addition to ECI, the procedure was supplemented by fibular grafting in most cases. Seven patients underwent vascular fibular grafting. Eight patients underwent nonvascular fibula strut grafting. Four patients did not undergo fibular grafting. The radiation dose was uniform in all cases, i.e., 50Gy. Figures 2A-2D show the follow-up of a patient who underwent this procedure.

**Figure 3:**
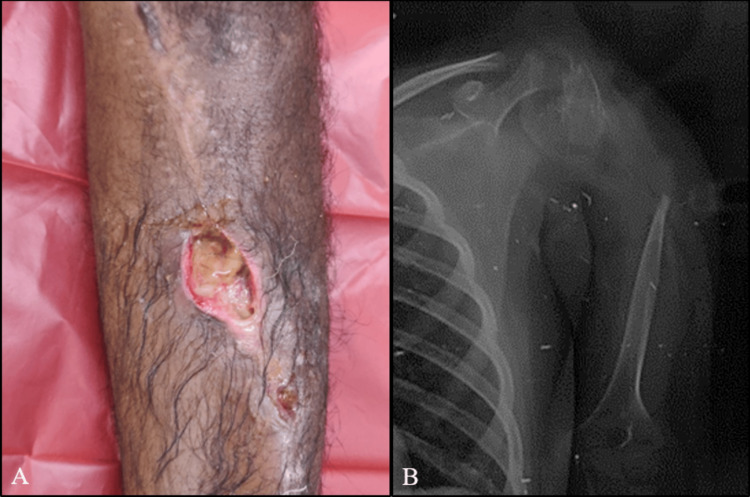
Challenging complications faced by us (A) Surgical site infection, (B) graft resorption

Union was seen in 18 cases by a maximum of seven months, with a mean duration of 4.75 months. The presence and type of fibular grafting did not seem to alter the union time. During the course of follow up, two patients (10.5%) were found to have local recurrence at six months and three months respectively. Amputation was performed in both these cases. Deep infection was a challenging complication encountered in four cases (Figure 3A). It was managed by Intravenous antibiotics, implant removal and debridement in three cases. As one of these three cases had fibrous union, an external fixator was placed till infection was resolved. She is currently being mobilized with the help of brace support as union is still in progress. One patient with chronic pus discharge, implant exposure and poor patient compliance underwent amputation. Limb length discrepancy was seen in two pediatric patients in whom the physis was sacrificed due to the extent of the tumor. One patient with proximal humerus reconstruction had gradual resorption of the irradiated bone after two years of surgery leading to loss of shoulder function (Figure 3B).

Table 2 summarizes the common complications faced.

**Table 2:** Summary of complications

Complications	Percentage
Infection	21%
Local recurrence	10.5%
Limb length discrepency	10.5%
Non union	5.2%
Graft Lysis	5.2%

We reviewed the existing literature to look for the incidence of complications in identical studies and found that similar complications were faced in other institutes as well. A comparison table was drawn to view the frequency of various complications seen with the procedure (Table 3).

**Table 3:** Complications reported by various studies NR = Not reported

Complication	Davidson et al. [8](n=50)	Poffyn et al. [14] (n=108)	Sharma et al. [11] (n=14)	Shah et al. [15] (n=15)	Hong et al. [16] (n=101)	Puri et al. [10] (n=12)	Uyttendaele et al. [17] (n=17)	Present study (n=19)
Local recurrence	8%	0.9%	21%	7%	5%	17%	12%	10.5%
Infection	6%	34%	14%	21%	3%	17%	12%	21%
Graft necrosis	2%	NR	NR	NR	1%	NR	12%	5%
Non union	18%	14%	NR	NR	NR	16%	NR	5%
Fracture	4%	0.9%	NR	NR	1%	NR	NR	NIL

## Discussion

Limb preservation has changed from being an exception to a standard practice in the management of primary bone tumors [16]. Reconstruction of large intercalary defects can be challenging in spite of the many available options including metal prosthesis, free fibular transfer, allograft, distraction osteogenesis, and ECI. Each of the above-mentioned procedures has its pros and cons. ECI and reimplantation of the irradiated bone are indicated where the rest of the procedures are not feasible. Complications like loosening and breakage with metallic prosthesis, donor site morbidity, graft failure and fracture associated with free fibular graft, and prolonged treatment duration for distraction osteogenesis made ECI a viable option and it is thus gaining popularity. Since 1968, when Spira and Lubin first reported on the use of ECI and reimplantation in malignant bone tumors [11,12], various studies have been published concerning this technique as a useful method of limb salvage. Reimplanting the irradiated tumor-bearing bone segment provides an anatomical size-matched graft that acts as a scaffold for creeping substitution and incorporation. This procedure, when reinforced by using a vascular or nonvascular fibular graft provides additional mechanical and biological stability along with early union [18]. It is a relatively low-cost technique as compared to tumor prostheses. In a comparative study between segmental allograft and extracorporeally irradiated autograft reconstruction, Chen et al. found a significantly lower rate of nonunion with ECI [12]. In their study, fractures have been reported in both groups. This can occur because of bone destruction by the tumor causing a decrease in the mechanical strength. We do not have any incidence of fractures in our study. This is probably because of the addition of fibular grafting which improved the mechanical strength of the construct along with good union, thus reducing the risk of fracture.

The overall cost of the procedure is much less than that demanded by an endoprosthetic replacement. Also, since this is a biological method of reconstruction with the patient’s own bone, this method is more easily accepted by the patient without much deliberation. The optimum dose of radiation should not only be enough to kill all the tumor cells and prevent recurrence but also not too high to threaten the physical integrity and growth factors, thus leading to graft failure and nonunion. In their study, Hong et al. used 50Gy of radiation and found no evidence of local recurrence or symptoms of graft failure [16]. In our study, we used 50Gy of irradiation given as a single fraction over 20-30 minutes. Resorption of the irradiated graft was noted in one patient, resulting in abnormal mobility and inability to perform shoulder functions. But we believe that it is not related to the radiation dose as all the cases in our study received the same dose. Uyttendaele et al. found similar complications in their study but found that the overall function was largely satisfactory after performing a secondary procedure [17].

Some of the reported complications of this procedure include local recurrence and wound infection. Puri et al., in their study of 12 patients, reported infection in two patients which were managed by immediate graft removal and nonbiological reconstruction [10]. In another study by Gunaseelan et al., the rate of infection with this procedure was reported to be 12% [19]. Even though we faced a higher rate of infection-related complications of around 21%, we were able to manage it with the help of antibiotics, debridement, and implant removal. Thus, infection can be much better managed while using this technique when compared to endoprosthesis, where removal of the prosthesis or amputation would be the outcome. In our study, three patients with infection were managed by implant removal and six weeks of antibiotic treatment. One patient had to undergo amputation due to an intractable infection and poor compliance. Two patients developed local recurrence and had to undergo amputation within five months of surgery. This rate of recurrence is comparable with other types of reconstruction techniques.

Limb length discrepancy occurred in our patients where the physis had to be sacrificed but did not cause significant disability. It was seen in the upper limb in two cases and was within acceptable limits. Muramatsu et al. found that the combined use of vascularized and irradiated bone autografts provides the cumulative advantage of mechanical endurance from the latter and biological properties of the former [18]. We did not find any significant difference in the union time and functional outcome between the cases where we used vascularized fibular autograft and nonvascular fibula strut graft. But addition of a fibular graft can improve the mechanical stability of the construct which we believe is the reason why we did not encounter any fractures in our study.

Overall, the patients in our study expressed satisfaction with the procedure and were able to perform all activities of daily living. There was no requirement for revision surgeries or redo procedures. This procedure has proved to be an acceptable method of reconstruction, whenever there is a limitation for other reconstructive options. Limitations of this study include a small sample size and a heterogeneous study population. A larger sample size and further follow-up are required to further study the complications associated with the technique and to gain knowledge on preventing them.

## Conclusions

Based on our experience, we would like to conclude that ECI is a satisfactory alternative to reconstructing the bone defects resulting from tumor resection. In spite of complications such as infection, limb length shortening, graft resorption, etc., patients seem satisfied with the overall outcome of the procedure. Also, it is an affordable procedure for patients with low economic conditions. Patient acceptance and satisfaction post tumor resection and ECI were found to be higher than any other procedure as they are getting their native bone back.
